# Surprisingly High Specificity of the PPD Skin Test for *M. tuberculosis* Infection from Recent Exposure in The Gambia

**DOI:** 10.1371/journal.pone.0000068

**Published:** 2006-12-20

**Authors:** Philip C. Hill, Roger H. Brookes, Annette Fox, Dolly Jackson-Sillah, Moses D. Lugos, David J. Jeffries, Simon A. Donkor, Richard A. Adegbola, Keith P. W. J. McAdam

**Affiliations:** Bacterial Diseases Programme, Medical Research Council Unit, Banjul, The Gambia; Columbia University, United States of America

## Abstract

**Background:**

Options for intervention against *Mycobacterium tuberculosis* infection are limited by the diagnostic tools available. The Purified Protein Derivative (PPD) skin test is thought to be non-specific, especially in tropical settings. We compared the PPD skin test with an ELISPOT test in The Gambia.

**Methodology/Principal Findings:**

Household contacts over six months of age of sputum smear positive TB cases and community controls were recruited. They underwent a PPD skin test and an ELISPOT test for the T cell response to PPD and ESAT-6/CFP10 antigens. Responsiveness to *M. tuberculosis* exposure was analysed according to sleeping proximity to an index case using logistic regression. 615 household contacts and 105 community controls were recruited. All three tests assessed increased significantly in positivity with increasing *M. tuberculosis* exposure, the PPD skin test most dramatically (OR 15.7; 95% CI 6.6–35.3). While the PPD skin test positivity continued to trend downwards in the community with increasing distance from a known case (61.9% to 14.3%), the PPD and ESAT-6/CFP-10 ELISPOT positivity did not. The PPD skin test was more in agreement with ESAT-6/CFP-10 ELISPOT (75%, p = 0.01) than the PPD ELISPOT (53%, p<0.0001). With increasing *M. tuberculosis* exposure, the proportion of ESAT-6/CFP-10 positive contacts who were PPD skin test positive increased (p<0.0001), and the proportion of ESAT-6/CFP-10 negative contacts that were PPD skin test negative decreased (p<0.0001); the converse did not occur.

**Conclusions/Significance:**

The PPD skin test has surprisingly high specificity for *M. tuberculosis* infection from recent exposure in The Gambia. In this setting, anti-tuberculous prophylaxis in PPD skin test positive individuals should be revisited.

## Introduction

Tuberculosis (TB) causes approximately 2 million deaths per year globally;[1] 98% of these occur in low-income countries.[2] New ways to tackle the epidemic in high prevalence, resource poor, settings are urgently needed.[3] One option is to develop interventions against *Mycobacterium tuberculosis* infection. The diagnosis of *M. tuberculosis* infection in tuberculosis (TB)-endemic tropical settings presents specific challenges. The abundance of environmental mycobacteria makes any test using purified protein derivative (PPD) potentially non-specific due to cross-reaction with *M. tuberculosis* antigens. It is also not possible to identify a sub-population that can be safely assumed not to be *M. tuberculosis* infected.

Recently we presented a reproducible model for assessing new diagnostic tests for *M. tuberculosis* infection in such a setting,[4], [5] a refinement of a model developed by Lienhardt et al.[6] Using an *ex vivo* ELISPOT assay that measures the precise IFN-γ T cell response to stimulatory antigens after an overnight incubation period, we identified that two *M. tuberculosis* antigens, ESAT-6 (6-kDa early secreted antigenic target) and CFP-10 (10-kDA culture fitrate protein) which are not found in BCG or many environmental mycobacteria, together offered improved specificity over PPD in the diagnosis of *M. tuberculosis* infection. However this appeared to be at the cost of some sensitivity when assessed against the traditional tuberculin skin test, which uses PPD. There was also poor agreement among the two ELISPOT measures and the PPD skin test. In the present study, we extended our exposure model into the community to define clearly how the PPD skin test, PPD ELISPOT test and the ESAT-6/CFP-10 ELISPOT test relate to each other across a complete gradient of recent exposure to a known TB case.

## Methods

### Participants

Sputum smear positive TB index cases over 15 years of age and their household contacts at least 6 months of age were recruited consecutively and selected for ELISPOT as previously described.[4] They were categorised according to where they slept as a proxy of *M. tuberculosis* exposure: in the same bedroom as the case, a different bedroom in the same house, or in a different house on the same compound.[4], [7]

Frequency matched community controls were recruited as follows. The age and sex of consecutively recruited TB case contacts were obtained from a previous case contact study in The Gambia and allocated to a newly recruited index case households. Index case households were asked if they were happy for a community control to be sought in the neighbourhood. Similar to a previous selection process in The Gambia[6] and other developing countries,[8] community controls were selected by choosing a random direction from the case's home (by spinning a pen in the air) and visiting the second compound on the right. The field-researchers checked that there was no history of a known TB case within the household and then asked whether there was anyone living the majority of the time in that compound of the required age band (5 year age bands for those under 15 years of age, 10 year age bands for those 15 years and older) and gender. If there was more than one possible match, the control was selected randomly by the toss of a coin or drawing blindly a numbered piece of paper from a container. If there was no possible control at that compound or refusal to participate, the process was repeated once more.

Household contacts and community controls underwent a PPD skin test (2 TU, PPD RT23, Statens Serum Institut, Copenhagen, Denmark) immediately after being bled. Induration was recorded at 48–72 hours by field staff blinded to the exposure category of the subject. Subjects with a positive skin test (mean of the longitudinal and transverse induration diameter ≥10 mm) were offered a chest X-ray and those with symptoms underwent a clinical assessment. Those with TB disease were referred to the National Programme for free treatment. There is no current practice of preventive treatment in The Gambia.

This study was approved by the joint Gambia Government/MRC Ethics Committee.

### Laboratory procedures

Sputum smears from TB index cases were prepared, stained, cultured, identified and confirmed as previously described.[9] The *ex-vivo* ELISPOT assays for IFN-γ were performed as previously described.[10] Purified Protein Derivative (*M. tuberculosis*, RT49, Statens Serum Institut, Copenhagen, Denmark) was used at 10 µg/ml. The positive control was Phytohaemaglutinin (PHA; Sigma-Aldrich, UK). All antigens were tested in duplicate wells.

ELISPOT plates were counted with an ELISPOT reader (AID-GmbH, Strassberg, Germany). The spot forming unit (SFU) numbers counted in each well were automatically entered into a database. Supplementary data were entered by double data entry. Positive test wells were pre-defined as containing at least ten SFUs more than, and at least twice as many as, negative control wells. PHA positive control wells were set to at least 150 SFUs above negative control wells. Negative control wells were required to have less than 30 SFUs. Laboratory staff were blinded as to the exposure category of the subject tested.

Testing for HIV-1 or HIV-2 infection was by competitive enzyme linked immunosorbent assays (Wellcome Laboratories, Kent, UK) and Western blot (Diagnostics Pasteur, Marnes-la-Coquette, France). HIV positive individuals were referred to a specialist clinic that now offers free anti-retroviral treatment according to set criteria.

### Data management and analysis

All data were entered using double data entry into a relational ACCESS database[11] and checked for errors. Random effects logistic regression models, taking into account household clustering, were used to assess the relationship between risk factors and test results and to provide summary p values for the comparison of the relative ESAT-6/CFP-10 ELISPOT and skin test proportions across the sleeping exposure gradient. Results were reported as unadjusted and adjusted odds ratios and their 95% confidence intervals (CI) and/or p values. Concordance between the tests was calculated using the Kappa statistic and discordance by McNemar's test. All statistical analyses were conducted using Stata software (version 7; Stata Corp, College Station, TX).

## Results

**Figure 1:**
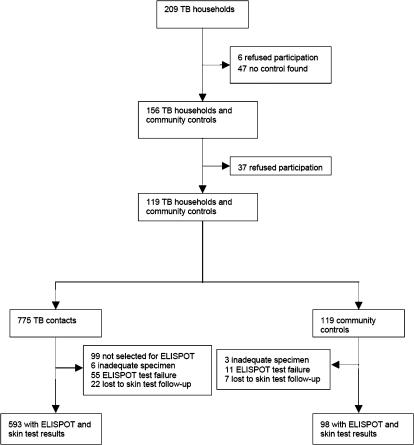
Study profile.

From 1 July 2002 to 9 February 2004, 209 index case households were recruited and had ELISPOT and skin test results (Figure 1). The final study population comprised 775 case contacts and 119 community controls. Of these, 720 were selected for, and had, ELISPOT results and six hundred and ninety one subjects had a PPD skin test read between 48 and 72 hours; 90 individuals had an independent second skin test reading for quality control and there was agreement with respect to a positive or negative result in 88 (98%). The characteristics of the community controls were similar to the contacts with respect to sex and HIV status, but were slightly older (mean 26.8 years vs 21.3 years; p = 0.0001) and a higher proportion had a BCG scar (59% vs 42%; p = 0.0012; Table 1).

**Table 1:**
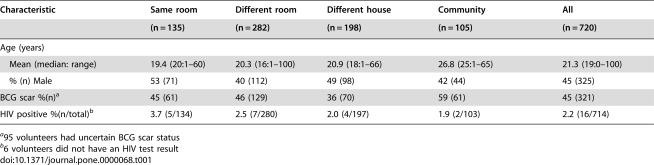
Characteristics of 720 TB case contacts and community controls by sleeping exposure category

Characteristic	Same room	Different room	Different house	Community	All
	(n = 135)	(n = 282)	(n = 198)	(n = 105)	(n = 720)
Age (years)					
Mean (median: range)	19.4 (20:1–60)	20.3 (16:1–100)	20.9 (18:1–66)	26.8 (25:1–65)	21.3 (19:0–100)
% (n) Male	53 (71)	40 (112)	49 (98)	42 (44)	45 (325)
BCG scar %(n)^a^	45 (61)	46 (129)	36 (70)	59 (61)	45 (321)
HIV positive %(n/total)^b^	3.7 (5/134)	2.5 (7/280)	2.0 (4/197)	1.9 (2/103)	2.2 (16/714)

a 95 volunteers had uncertain BCG scar status

b 6 volunteers did not have an HIV test result

**Figure 2:**
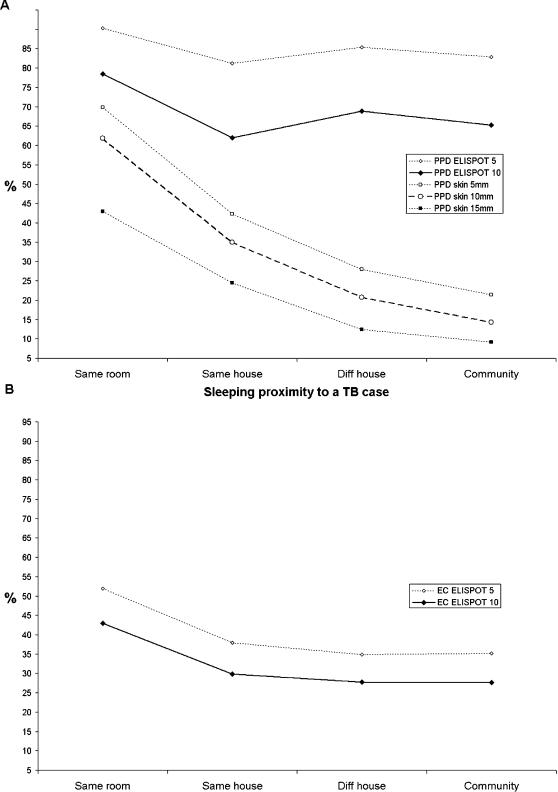
Percent positive for each test by exposure category. **A.** PPD ELISPOT positive and percent PPD skin test positive for those with a result for both tests (n = 691). PPD ELISPOT positivity is represented according to a predetermined 10 spot cut-off (PPD ELISPOT 10) as well as an alternative 5 spot cut-off (PPD ELISPOT 5). PPD skin test positivity is represented according to a predetermined 10 mm induration cut-off as well as 2 alternative cutoffs: 5 mm and 15 mm. **B.** ESAT-6/CFP-10 ELISPOT positivity represented according to a predetermined 10 spot cut-off (EC ELISPOT 10) as well as an alternative 5 spot cut-off (EC ELISPOT 5).

Overall 228 (33.0%) of 691 contacts tested were PPD skin test positive, 226 (31.4%) and 489 (67.9%) of 720 tested were ELISPPOT positive for ESAT-6/CFP-10 and PPD respectively. The percentage of volunteers positive increased significantly from the community to the bedroom of a known TB index case for all three tests, most markedly for the PPD skin test (OR 15.7, 95% CI 6.6–35.3; Table 2) and none of the tests was confounded by the presence of a BCG scar when introduced into the logistic regression model. Figure 2 shows the percentage of individuals positive for each test across the exposure gradient for all those with both ELISPOT and skin test results. Even with a conservative 10 spot cut-off used in this study, PPD ELISPOT positivity was over 60% in all exposure categories. Using an alternative 5-spot cut-off, PPD ELISPOT positivity ranged from 81% to 90%. In contrast PPD skin test positivity was 62% in the highest exposure category and trended downwards to reach only 14% in the community. This trend occurred irrespective of whether a 5, 10 or 15 mm PPD skin test cut-off was considered. The ESAT-6/CFP-10 ELISPOT (Figure 2B) responded to the exposure gradient more dramatically than the PPD ELISPOT, but less dramatically than the PPD skin test: the proportion positive by skin test was higher in the highest exposure category and lower in the community. This finding held true when considered separately in 3 age groups: under 10 years old, those aged 10–30 and those over 30 years old (data not shown).

**Table 2:**
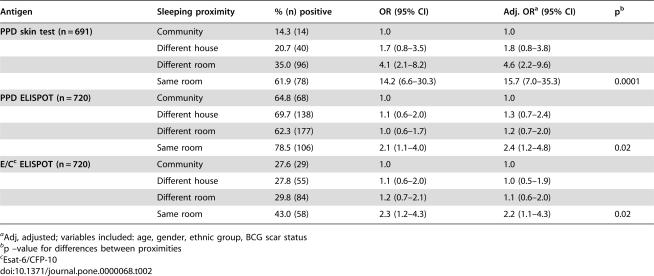
Univariable and multivariable odds ratios for the ELISPOT and PPD skin tests by logistic regression (household as a random effect) according to sleeping proximity to a case

Antigen	Sleeping proximity	% (n) positive	OR (95% CI)	Adj. OR^a^ (95% CI)	p^b^
**PPD skin test (n = 691)**	Community	14.3 (14)	1.0	1.0	
	Different house	20.7 (40)	1.7 (0.8–3.5)	1.8 (0.8–3.8)	
	Different room	35.0 (96)	4.1 (2.1–8.2)	4.6 (2.2–9.6)	
	Same room	61.9 (78)	14.2 (6.6–30.3)	15.7 (7.0–35.3)	0.0001
**PPD ELISPOT (n = 720)**	Community	64.8 (68)	1.0	1.0	
	Different house	69.7 (138)	1.1 (0.6–2.0)	1.3 (0.7–2.4)	
	Different room	62.3 (177)	1.0 (0.6–1.7)	1.2 (0.7–2.0)	
	Same room	78.5 (106)	2.1 (1.1–4.0)	2.4 (1.2–4.8)	0.02
**E/C** c **ELISPOT (n = 720)**	Community	27.6 (29)	1.0	1.0	
	Different house	27.8 (55)	1.1 (0.6–2.0)	1.0 (0.5–1.9)	
	Different room	29.8 (84)	1.2 (0.7–2.1)	1.1 (0.6–2.0)	
	Same room	43.0 (58)	2.3 (1.2–4.3)	2.2 (1.1–4.3)	0.02

a Adj, adjusted; variables included: age, gender, ethnic group, BCG scar status

b p –value for differences between proximities

c Esat-6/CFP-10

**Figure 3:**
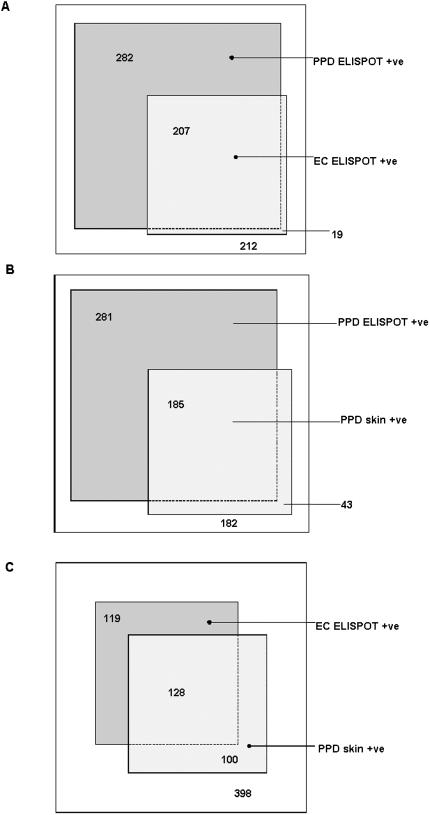
Scaled rectangular diagrams showing relative proportions of TB case contacts who are PPD skin test positive and/or ELISPOT test result positive in relation to each other. The sizes of the boxes are proportional to the relative number of individuals they represent and the numbers represent numbers of individuals. **A.** PPD ELISPOT and ESAT-6/CFP-10 (EC) ELISPOT (n = 720) **B.** PPD ELISPOT and PPD skin test (n = 691) **C.** ESAT-6/CFP-10 ELISPOT and PPD skin test (n = 691)

**Figure 4:**
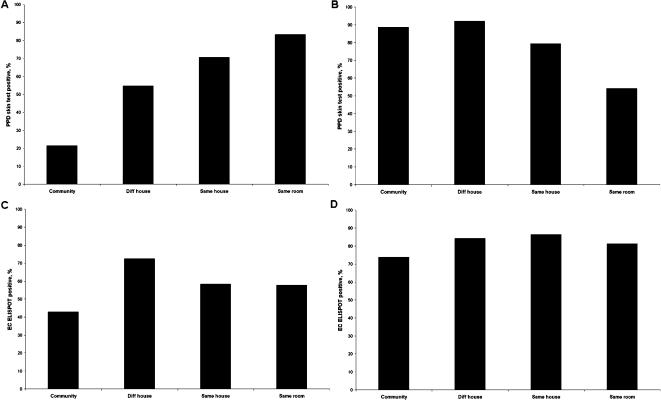
Comparison of PPD skin test and ESAT-6/CFP-10 (EC) ELISPOT test in relation to each other, expressed as percentages across exposure category by sleeping proximity to a TB case. **A.** Proportion of ESAT-6/CFP-10 positive who are PPD skin test positive (n = 215) **B.** Proportion of ESAT-6/CFP-10 negative who are PPD skin test negative (n = 476) **C.** Proportion of PPD skin test positive who are ESAT-6/CFP-10 positive (n = 228) **D.** Proportion of PPD skin test negative who are ESAT-6/CFP-10 negative (n = 463)

Figure 3 shows the frequencies of a positive result for each test in relation to each other by the use of scaled rectangle diagrams, with areas of overlap proportional to frequency.[12] The overall agreement was 53% between PPD ELISPOT and PPD skin (concordance, Κ = 0.16; discordance, p<0.0001), 58% between PPD ELISPOT and ESAT-6/CFP-10 ELISPOT (concordance, Κ = 0.26; discordance, p<0.0001), but 75% between ESAT-6/CFP-10 ELISPOT and PPD skin (concordance, Κ = 0.43; discordance, p = 0 01). Noting the relative concordance between the PPD skin test and the ESAT-6/CF-10 ELISPOT, we assessed these two tests against each other in relation to the gradient of exposure by sleeping proximity to a case (Figure 4). It can be assumed that if test 1 is better than test 2, then the proportion of test 2 positive individuals who are test 1 positive should increase with increasing prevalence of true infection. Similarly, the proportion of test 2 negative individuals that are test 2 negative would be expected to decrease with increasing prevalence of true infection. Indeed, the proportion of ESAT-6/CFP-10 positive individuals that were PPD skin test positive increased significantly with increasing exposure to an index case (Figure 4A, p<0.0001) and the proportion of ESAT-6/CFP-10 negative individuals that were PPD skin test negative decreased (Figure 4B, p<0.0001). In contrast, the proportion of PPD skin test positive individuals that were ESAT-6/CFP-10 positive did not increase significantly with increasing exposure (Figure 4C, p = 0.10) and the proportion of PPD skin test negative individuals that were ESAT-6/CFP-10 negative actually increased slightly with increasing exposure (Figure 4D, p = 0.31).

## Discussion

This study has taken advantage of a reproducible gradient of exposure to *M. tuberculosis* in a TB-endemic tropical setting and extended it into the community. The comparison of PPD skin test versus PPD ELISPOT revealed what one would expect when comparing a specific test with a non-specific one across different sub-populations with varying prevalence of true infection. Indeed the PPD skin test was more in agreement with the ELISPOT assay employing more specific *M. tuberculosis* antigens. When the PPD skin test and the ESAT-6/CFP-10 ELISPOT were compared with each other across the exposure gradient, the PPD skin test related to the gradient more accurately. These results provide important insights into the different properties of the skin test and T cell response to mycobacterial antigens.

The finding of high specificity of the PPD skin test in settings such as The Gambia is supported by a recent review by Farhat et al.[13] Poor agreement between T cell and skin test responses to PPD has been reported elsewhere in Africa.[14] It has also been shown to vary by latitude.[15] In addition, rapid reversion of a positive PPD skin test result after BCG vaccination occurs in tropical settings while it is more likely to be persistent in more temperate locations and when given at an older age.[16], [17] In relation to this, the PPD skin test in The Gambia and many other tropical settings is not affected by prior BCG vaccination, even in young children.[4], [7] These observations and the findings of the present study may have a unifying explanation. Hoft et al[18] have shown that regular mucosal exposure to BCG leads to suppression of skin test responses but a significant increase in mycobacteria-specific IFN-γ responses in peripheral blood. Therefore, frequent oral ingestion of non-pathogenic environmental mycobacteria in TB-endemic tropical settings could result in unexpected loss of PPD skin test reactivity over time, poor agreement between skin test and peripheral blood responses to mycobacterial antigens and surprisingly high PPD skin test specificity. Compartmentalisation of the antigen-specific immune response has been well-described in other situations and is likely to be mediated through lymphocyte homing in response to adhesion molecules and chemokines.[19]

There is also evidence from large trials of BCG vaccine[20] that an initial positive PPD skin test response is much more specific for *M. tuberculosis* infection than one that occurs only as a result of a 2-step procedure. Those positive by initial PPD skin test or through a 2-step procedure, were sometimes followed in parallel with those who were PPD skin test negative that were entered into the trials. The MRC tuberculosis vaccines clinical trials committee presented such a follow-up of 56,000 adolescents in 1956.[21] The incidence rate of definite TB disease in those initially skin test positive was 175/100,000 per year compared to 74/100,000 per year in those only positive in a two-step test. Similarly, in 1969, Comstock and Webster reported a 20 year follow-up of school children in Georgia, where 29/1492 (2%) of those initially positive became definite TB cases versus 7/3768 (0.2%) of those positive through a two-step procedure.[22]

Studies of T cell assays that employ more ESAT-6 and CFP-10 have compared them to the PPD skin test.[23], [24] However, it has been unclear what proportion of the differences, or similarities, seen are due to the antigens versus the different immune responses being evaluated. While the use of ESAT-6 and CFP-10 improves specificity within the ELISPOT assay, this is at the cost of some sensitivity in The Gambia when compared to the PPD skin test,[4] even when a 5-spot cut-off is used (Figure 2B). We have also found that the ELISPOT response to ESAT-6 varies according to infecting *M. tuberculosis* strain.[25] Furthermore, it is known that ESAT-6 and CFP-10 are secreted by some environmental mycobacteria commonly encountered in The Gambia,[26], [27] potentially compromising test specificity in our setting.

Do the findings of the present study provide evidence that the PPD skin test is actually both more sensitive and specific than the ESAT-6/CFP-10 ELISPOT for *M. tuberculosis* infection in The Gambia? The answer to that question is ‘yes and no’. It certainly appears clear that, with respect to new infection from recent exposure, the PPD skin test is more sensitive than the ESAT-6/CFP-10 ELISPOT and also more specific. However, with respect to latent infection from more distant exposure, the PPD skin test appears to be heavily down-regulated in this TB-endemic setting as it seems likely that more than 14% of the community are harbouring *M. tuberculosis*. Therefore the interpretation of a positive or negative ESAT-6/CFP-10 ELISPOT in the community in The Gambia is complex. Longitudinal follow-up to identify secondary cases will be important in trying to resolve this issue.

That the PPD skin test is negative in some individuals who may have latent *M. tuberculosis* infection is of particular concern in certain subgroups, such as those who are immunosuppressed, where there may be a niche for new generation T cell assays in Africa.[28] It is of particular relevance in HIV positive individuals with advanced immunodeficiency and for those planning safety studies of new generation TB vaccines, as it is feared that highly immunogenic vaccines may induce a ‘Koch phenomenon’ in those with latent *M. tuberculosis* infection.[29] In our new generation TB vaccine safety studies in The Gambia, we have introduced both ELISPOT and PPD skin test entry criteria for volunteers recruited from the general community.

Our study has several possible sources of bias. First, refusal to participate by some case households and a number of potential community controls was understandable as TB cases may be afraid that neighbours would find out about their disease and individuals in the community are often reluctant to be bled. We note that the characteristics and test results of the households in this study were similar to those of the households in our previous study.[4] Second, the community controls were slightly older than the household contacts. Age standardised ELISPOT and skin test positivity were not different from the crude results however (data not shown) and one would expect older age to only increase the proportion of individuals who are PPD skin test positive in a TB-endemic setting.[17] Third, a higher proportion of community controls had a BCG scar. While there is no significant effect of the BCG scar on the PPD skin test or ELISPOT results in The Gambia,[4] it could only be expected to result in increased positivity by PPD skin test in these individuals.

The highest risk group for progression to TB are those most recently infected, their risk being greatest in the first year after exposure.[30] The results of this study suggest that the PPD skin test has surprisingly high specificity with respect to this group in our setting and is otherwise likely to be under anergic pressure, becoming positive upon exposure to true pathogen. These findings have important public health implications. There is evidence that preventive therapy had a larger impact in the control of the TB epidemic in the United Kingdom than either vaccination or chemotherapy.[31] High PPD skin test specificity in The Gambia opens up new possibilities to revisit strategies to control the TB epidemic that include prophylaxis against the development of disease at a population level. In our setting, 3% of PPD skin test positive case contacts at screening become TB cases within 1 year, versus 1% of those who are PPD skin test negative (unpublished data). Furthermore, up to 45% of new TB cases report household contact with a known TB case.[32] Therefore, preventive therapy in PPD skin test positive TB case contacts may be an appropriate and important first step.
